# The pro-fibrogenic role of SPP1^+^ macrophages in medical implant fibrosis: mechanisms and therapeutic opportunities

**DOI:** 10.3389/fimmu.2026.1749098

**Published:** 2026-02-03

**Authors:** Mei Yu, Jiayin Fu

**Affiliations:** 1 The First People’s Hospital of Xiaoshan District, Xiaoshan Affiliated Hospital of Wenzhou Medical University, Hangzhou, China; 2 Department of Cardiology, Sir Run Run Shaw Hospital, Zhejiang University School of Medicine, Hangzhou, China

**Keywords:** extra cellular matrix, implant fibrosis, lipid-associated macrophages, macrophage, SPP1^+^

## Abstract

Fibrosis is a major cause of biomedical device failure. Recent advances identify SPP1^+^ macrophages as pivotal regulators of this process. These cells derive from CCR2^+^ monocytes, adopt a lipid-associated macrophage-like phenotype, and terminally differentiate within collagen-rich niches at implant interfaces. Spatiotemporal analysis reveals their recruitment via the CCL2-CCR2 axis, persistence in chronic phases (>4 weeks), and spatial co-localization with activated fibroblasts at fibrotic fronts. Through osteopontin-CD44 signaling, they drive fibroblast-to-myofibroblast differentiation and pathological extracellular matrix deposition. Building on this mechanistic insight, emerging therapeutic strategies specifically targeting SPP1^+^ macrophages, such as blockade of the CCL2-CCR2 axis, inhibition of osteopontin-CD44 signaling, CRISPR-Cas13-based gene circuits, engineered CAR macrophages, and smart biomaterial-based drug delivery systems, hold great promise for mitigating implant-associated fibrosis. A comprehensive understanding of the role of SPP1^+^ macrophages, coupled with these novel interventions, is crucial for developing precise antifibrotic therapies to maintain the long-term functionality of medical implants.

## 1 Introduction

Fibrosis, a pathological accumulation of extracellular matrix (ECM) leading to tissue scarring, is a major reason for implant failure in biomedical devices, including artificial vascular implants, pacemakers, joint prostheses, and biosensors. This process is driven by chronic inflammation triggered by foreign−body responses, where macrophages play a central role in orchestrating fibrotic cascades (1, 2). Recent advances in single−cell transcriptomics have revealed that a distinct subset of macrophages, characterized by high expression of secreted phosphoprotein 1 (SPP1, encoding osteopontin), is intimately implicated in fibrosis across multiple organs (e.g., heart, liver, lungs, and skin) (3–5). SPP1^+^ macrophages typically derive from C−C motif chemokine receptor 2 (CCR2)−positive monocytes and exhibit a lipid−associated macrophage (LAM)−like transcriptional profile (co−expressing TREM2, CD9, APOE) (6–11). In fibrotic niches, these cells localize at the periphery of collagen−rich scars and interact directly with activated fibroblasts and myofibroblasts (12). Through secretion of osteopontin (SPP1), fibronectin, and semaphorins, SPP1^+^ macrophages promote fibroblast−to−myofibroblast differentiation and enhance pathological ECM deposition (13, 14). Notably, their accumulation correlates with progressive fibrosis in clinical conditions such as idiopathic pulmonary fibrosis (IPF) (15), liver cirrhosis (16), and systemic sclerosis (17).

In the context of implant fibrosis, SPP1^+^ macrophages contribute to peri−implant fibrotic capsule formation (5), a key reason for device dysfunction. Their spatial enrichment near collagenous matrices and temporal persistence during chronic inflammation suggest they drive sustained fibroblast activation around implants (18). Moreover, SPP1^+^ macrophage−derived osteopontin aggravates ECM production and tissue stiffening by fibroblasts via CD44 (19), which further compromise implant integration (20, 21). Therapeutic strategies targeting SPP1^+^ macrophage recruitment (e.g., blocking CCR2) (22) or their function (e.g., inhibiting osteopontin-CD44 signaling) (19) have shown promise in mitigating organ fibrosis and may also be applicable to implant−related fibrosis. This review discusses the spatiotemporal dynamics of SPP1^+^ macrophages in implant fibrosis, highlighting their role as pivotal regulators of fibroblast crosstalk, ECM remodeling, and pathological scarring. Understanding their recruitment, differentiation, niche−specific activation, and function in fibrosis may inspire novel anti−fibrotic therapies to extend the longevity of implants.

## 2 Spatiotemporal dynamics and molecular mechanisms of SPP1^+^ macrophages in implant fibrosis

**Figure 1:**
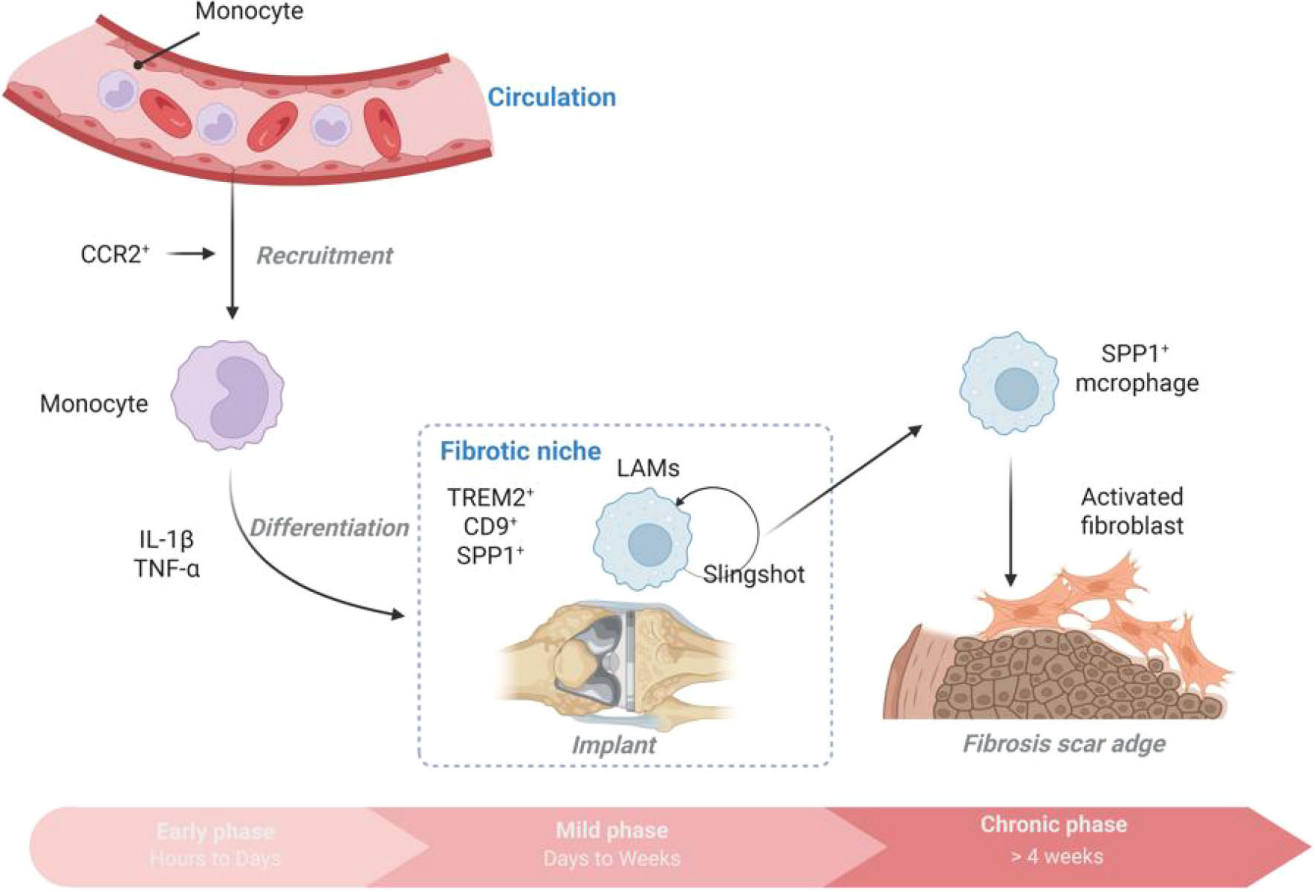
Time dynamics of SPP1^+^ macrophages after implantation.

SPP1^+^ macrophages are key regulators of implant fibrosis, following a spatiotemporal dynamic trajectory of “monocyte → LAM transition → SPP1^+^ terminal differentiation.” They can drive fibrosis through multiple axes, including the SPP1−CD44/TGF−β/MMP axis, YAP/TAZ pathway, integrin−ECM signaling, and β−catenin signaling. Targeting their differentiation or signaling pathways may alleviate fibrous encapsulation around implants (as shown in **Figure 1**).

### 2.1 Time dynamics

In the early stage after implantation (hours to days), monocytes (Ly6C^hi^/CCR2^+^) are recruited to the injury site and differentiate into pro-inflammatory macrophages (secreting IL-1β, TNF-α) (23). Over time (days to weeks), monocyte-derived macrophages differentiate towards a LAM state and begin to express markers such as TREM2 and CD9 (24, 25). This transitional LAM phenotype, characterized by a distinct lipid-handling transcriptional profile, has been identified as a key intermediate not only in fibrosis but also in contexts like myocardial ischemia-reperfusion injury (6). Within the chronic inflammatory milieu (>4 weeks post−implantation), LAMs further differentiate into SPP1^+^ macrophages (highly expressing osteopontin), which localize at the edge of fibrous scars and co−localize with activated fibroblasts (8). During the chronic inflammatory phase (7 months in humans and 28 days in mice after implantation), SPP1^+^ macrophages become the predominant cells promoting fibrosis (11).

### 2.2 Space distribution

SPP1^+^ macrophages are not diffusely distributed but are spatially constrained within the fibrotic niche. They preferentially accumulate within collagen−rich areas at the implant−tissue interface, forming close spatial associations with activated α−SMA^+^ myofibroblasts (8, 11). For instance, in silicone implant models, SPP1^+^ macrophages aggregate at the acellular dermal matrix (ADM) capsule interface, engaging in direct cellular crosstalk with fibroblasts and CD4^+^ T cells (11). Spatial transcriptomics reveals their enrichment at the fibrotic front (such as the portal vein area in liver fibrosis and the alveolar septa in pulmonary fibrosis), forming a “pro-fibrotic niche” (8). This pattern of spatially restricted, niche-resident macrophage activation is a recurring theme in tissue repair, as similarly observed in liver regeneration where specific macrophage subsets are anatomically confined to support reparative processes (7).

### 2.3 Differentiation of SPP1^+^ macrophages from CCR2^+^ monocyte

The CCL2−CCR2 axis mediates the recruitment of monocytes from bone marrow to implantation sites (26). Platelet−derived CXCL4 drives the differentiation of monocytes toward the SPP1^+^ phenotype (5, 8). TGF−β1 and hypoxia induce SPP1 expression through HIF−1α, promoting the transformation of macrophages toward a pro−fibrotic phenotype (8, 18). SPI1 (PU.1) and MYC transcription factors are upregulated, activating SPP1 and related ECM regulatory genes, such as matrix metalloproteinase 9 (MMP9) and tissue inhibitor of metalloproteinases 1 (TIMP1) (8, 27).

## 3 The role of SPP1^+^ macrophages in implant-related fibrosis

SPP1^+^ macrophages play a central regulatory role in implant−related fibrosis, driving irreversible pathological scar formation through fibroblast crosstalk, ECM remodeling, and construction of a pro−fibrotic microenvironment.

### 3.1 Fibroblast crosstalk: SPP1^+^ macrophages as the “fibrosis signaling hub”

SPP1^+^ macrophages activate fibroblasts through direct contact and paracrine signaling, promoting their differentiation into myofibroblasts. Osteopontin secreted by macrophages binds to CD44 and αvβ3 integrins on the surface of fibroblasts, activating the FAK−Src−ERK signaling pathway and promoting fibroblast proliferation and α−SMA expression (8). The centrality of this SPP1−CD44 axis is strongly supported by evidence from human fibrotic diseases; it is a key driver of collagen deposition in systemic sclerosis (28), and its elevated expression correlates with poor prognosis in idiopathic pulmonary fibrosis (19). SPP1^+^ macrophages secrete TGF−β1 and PDGF−AA, which, in conjunction with the TGF−βR/Smad pathway in fibroblasts, induce the expression of COL1A1/COL3A1 genes (8). In a mouse model of myocardial fibrosis, macrophage-specific knockout of TGF-β1 reduces collagen deposition by 50% (29). Additional crosstalk mechanisms include the activation of fibroblast β-catenin signaling via macrophage-derived WNT ligands (e.g., WNT5A), a pathway implicated in chronic kidney disease fibrosis (30).

### 3.2 ECM remodeling: from degradation imbalance to pathological deposition

SPP1^+^ macrophages promote pathological ECM remodeling and facilitate fibrotic ECM regulation by regulating the imbalance of matrix synthesis and degradation. They provide a stent for collagen fibers by upregulating fibronectin (FN1) and laminin (LAMB1). They secrete lysyl oxidase (LOX) to promote collagen cross-linking and strength of the ECM (8). High expression of MMP9/MMP12 degrades normal basement membranes (such as type IV collagen), while inhibiting TIMP1/2 prevents ECM degradation, leading to scar formation and hardening (31), which is a manifestation of protease-inhibitor imbalance. In lung tissues of patients with IPF, an elevated ratio of MMP9/TIMP1 in SPP1^+^ macrophage is positively correlated with disease progression (15).

### 3.3 “Positive feedback loop” of pathological scars

**Figure 2:**
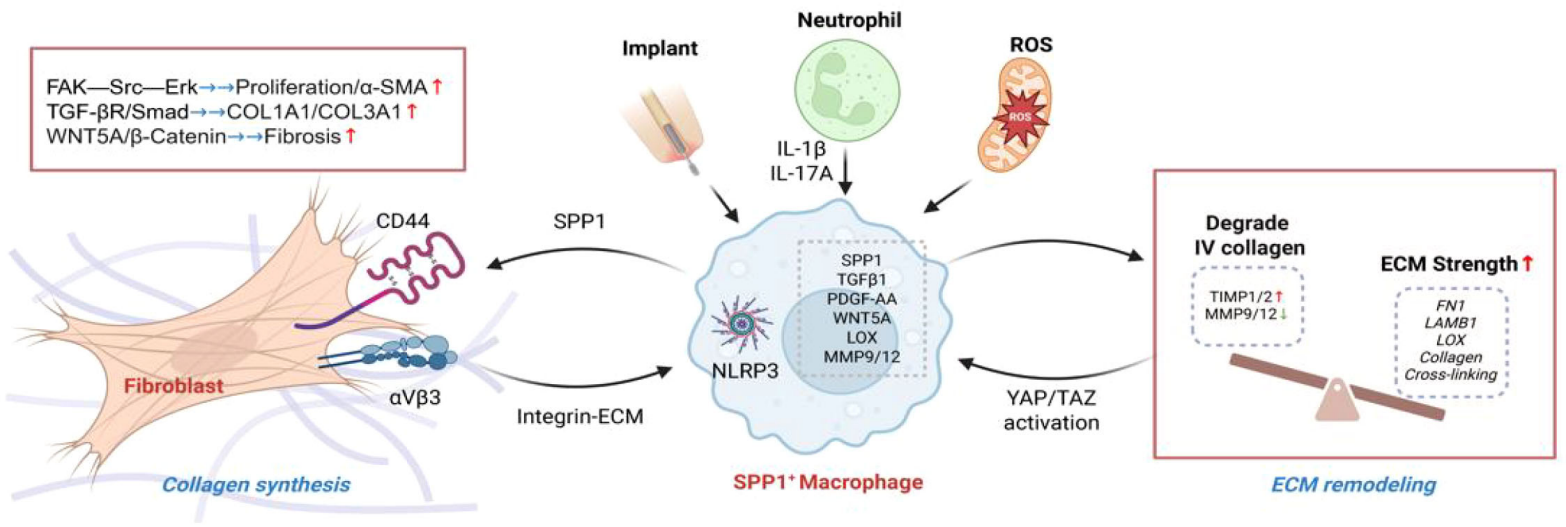
SPP1^+^ macrophages in implant-related fibrosis.

SPP1^+^ macrophages and the fibrotic microenvironment form a self-reinforcing network: inflammation-fibrosis coupling. Neutrophils are recruited through IL-1β/IL-17A, and they release reactive oxygen species (ROS) to further activate macrophages (32). Around the implant, we speculate that continuous foreign body stimulation leads to the activation of the NLRP3 inflammasome, maintaining the polarization of SPP1^+^ macrophages (33). The sclerotic ECM enhances the pro-fibrotic phenotype of macrophages through the YAP/TAZ pathway (34). The increased contractility of fibroblasts amplifies the feedback through integrin-ECM signaling, further recruiting SPP1^+^ macrophages (8). (As shown in **Figure 2**)

## 4 Therapeutic strategies targeting SPP1^+^ macrophage

SPP1^+^ macrophages play a critical role in fibrosis around implants, where they contribute to dense fibrous capsule formation, foreign−body reactions, and eventual implant failure (11, 35). To address this, emerging biomaterial−based therapies are being developed to specifically regulate SPP1^+^ macrophage activity (36). For example, ADM, used clinically with silicone breast implants, exerts its anti−fibrotic effect by upregulating SPP1. In both human and mouse models, elevated SPP1 levels correlated with an attenuated foreign−body response and reduced fibrotic capsule formation. Polymer nanoparticle (PNP) composites can deliver recombinant SPP1 protein in a controlled manner, significantly reducing collagen deposition around implants (11). SPP1^+^ macrophages exhibit a unique anti−fibrotic phenotype, characterized by high expression of MMP9 and downregulation of pro−inflammatory cytokines like IL−1β and TNF−α. Their activity is mediated through the HIF−1 and VEGF signaling pathways, which promote angiogenesis and tissue repair (37). Specifically, SPP1 binding to CD44 receptors on macrophages and fibroblasts upregulates MMP9, enhancing collagen degradation and preventing excessive ECM deposition (11, 38–40). Further studies indicate that SPP1^+^ macrophages suppress the transition of M1 macrophages toward a pro−fibrotic phenotype while promoting anti−inflammatory M2 polarization (41). SPP1 upregulation is associated with epigenetic modifications, such as H3K27ac enrichment, suggesting that its anti−fibrotic effects may be sustained through histone remodeling (42). Conversely, titanium nanoparticles (TiO^2^ NPs) activate SPP1 transcription, which in turn promotes fibrosis around titanium implants. This pro−fibrotic effect is mediated by SNAI2−driven SPP1 expression, which activates the PI3K/AKT pathway, leading to collagen deposition and myofibroblast differentiation (43).

**Figure 3:**
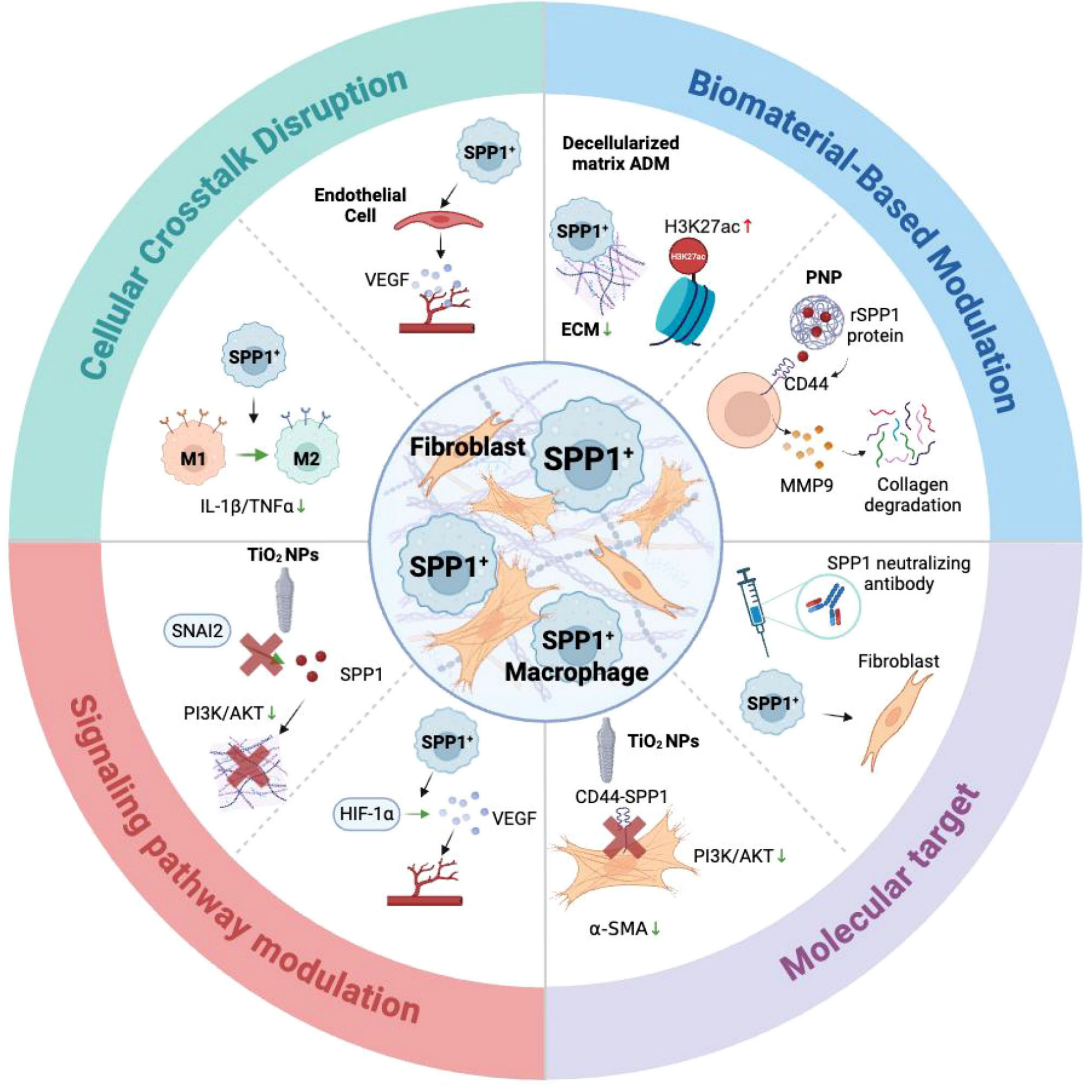
Therapeutic strategies targeting SPP1^+^ macrophage to implant-related fibrosis.

The most direct strategy is to inhibit the SPP1−CD44 signaling axis between macrophages and fibroblasts. Neutralizing antibodies against SPP1 or small−molecule inhibitors of CD44 have shown efficacy in reducing fibrosis in models of systemic sclerosis and non−alcoholic fatty liver disease (28, 44, 45). To prevent the initial accumulation of SPP1^+^ macrophage precursors, blockade of the CCL2-CCR2 axis remains a key strategy to inhibit monocyte recruitment to the implant site (26, 35). CRISPR-Cas13 dynamic regulatory loop construction “α-SMA biosensor-Cas13” gene circuit to automatically downregulate SPP1 expression when the fibrosis marker (α-SMA) is elevated. CAR macrophage therapy for engineered macrophages expressing antifibrotic receptors reduces the fibrotic area by 40% in a myocardial injury model, and is expected to be used in the treatment of implant fibrosis (46). PNP delivery system can also load other drugs that regulate SPP1^+^ macrophages to treat implant fibrosis. SPP1^+^ macrophage can also be reprogramed via epigenetic regulation, such as histone modification or DNA methylation (47). Biomaterials that inhibit mechanical signaling have been shown to block the positive feedback loop between ECM sclerosis and macrophage activation (48, 49). (As shown in **Figure 3**)

## 5 Summary

Recent advances in single-cell transcriptomics and spatial multi-omics have revealed that SPP1^+^ macrophages are not a uniform population but display significant heterogeneity and tissue-dependent activation states during implant fibrosis. Originating from CCR2^+^ monocytes, these macrophages ultimately differentiate into the SPP1^+^ phenotype, colonizing collagen-rich regions at the implant-tissue interface. There, they co-localize with α-SMA^+^ myofibroblasts to establish a “pro-fibrotic microenvironment.” Within this niche, SPP1^+^ macrophages drive fibroblast activation and pathological extracellular matrix deposition primarily through the osteopontin-CD44 signaling axis (11, 40, 44). These terminally differentiated macrophages perpetuate chronic fibrotic responses by secreting fibronectin, MMPs, and TGF-β (8, 50). SPP1^+^ macrophages, as the core effector cells of implant fibrosis, provide a new paradigm for anti-fibrotic therapy by analyzing their spatiotemporal dynamics and molecular mechanisms. In the future, it is necessary to integrate intelligent biomaterials, gene editing, and precise interventions guided by multi-omics to develop microenvironment responsive therapies, ultimately achieving a breakthrough in extending the functional lifespan of implants.
